# The association between dental caries and television viewing among Chinese adolescents in Guangxi, China

**DOI:** 10.1186/1472-6831-14-138

**Published:** 2014-11-25

**Authors:** Xiaojuan Zeng, Aubrey Sheiham, Wael Sabbah

**Affiliations:** College of Stomatology, Guangxi Medical University, Guangxi, China; Department of Epidemiology & Public Health, University College London, London, WC1E 6BT UK; Dental Institute, King’s College London, London, UK

**Keywords:** Dental caries, Adolescents, Television viewing, China

## Abstract

**Background:**

Television viewing has been implicated as a possible risk factor for the increase in a number of chronic diseases, particularly those related to sedentary life style. Given the rapid economic and societal changes in China over the past few decades, this study aimed to examine the association between dental caries experience and television viewing among Chinese adolescents.

**Methods:**

This study utilized data pertaining to the province of Guangxi from the 2010 National Physical Fitness and Health Surveillance, a national survey of school children and adolescents in China. The survey used stratified sampling methods. Four experienced dentists conducted the clinical examination in each province. The survey included data on socio-demographic and behavioural factors including television viewing and a clinical dental examination. Regression models were used to examine the association between time spent viewing television and mean DMFT and untreated caries among 12–17 year-old adolescents, adjusting for age, sex, ethnicity, area of residence, and markers of dietary habits.

**Results:**

The prevalence of caries in this sample was 52.3%. Longer duration of television viewing was significantly and consistently associated with greater number for decayed teeth and higher DMFT among Chinese adolescents. The relationship persisted even after adjusting for demographic and behavioural factors. Being female, living in rural area, being of Zhuang ethnicity were all significantly associated with higher levels of dental caries.

**Conclusions:**

This study showed, for the first time in China, that television viewing is associated with risk of developing dental caries among adolescents. Future research should examine potential pathways linking television viewing and dental caries among Chinese adolescents.

## Background

The rapid economic development in China over the past few decades has been accompanied by changes in behavioural patterns, lifestyle and cultural norms. As a result of those changes, there is a more sedentary lifestyle and changes in dietary habits to Western diets [1–3]. Consequently, the Chinese population is experiencing an epidemiological transition characterized by increasing prevalence of non-communicable diseases [2, 4]. The shifting behavioural patterns, particularly those related to diet, have resulted in an increase in the prevalence of dental caries among Chinese children and adolescents. The prevalence of caries for 7, 9, 12, 14, and 17 years was 2.3%, 5.6%, 13.9%, 19.0% and 21.2% respectively, among urban boys [5].

Epidemiological studies have identified television viewing as a possible risk factor for chronic health conditions that are associated with sedentary lifestyle and nutrition intake [6, 7]. Different pathways have been postulated for the relationship between TV viewing and poorer health [8]. First, longer hours spent watching TV leads to a reduction in physical activities and an increase in consumption of unhealthy snacks while watching TV [9]. Second, TV food advertisements affect food choices [10, 11].

Several studies reported that the frequency and the time of food advertisements on TV were directly linked to increased consumption of obesogenic foods and an increase in related health conditions [12]. A number of studies in Western and Asian countries argued that TV advertisements could have a similar impact on dental caries. For example 56% of food advertisement in England and 50% in India were about high sugar foods/beverages [13–17]. Given the recent and rapid economic and social changes in China accompanied by an increase in urbanization [1, 3], increase in the availability of Western foods and longer hours spent watching TV and/or using computers, we set out to examine whether TV viewing was related to dental caries in China. The objective of this study was to examine the association between dental caries experience and TV viewing among Chinese adolescents.

## Methods

Data for this analysis was derived from the 2010 National Physical Fitness and Health Surveillance, a national survey of school children and adolescents in China [18]. The Department of Education of Guangxi and Guangxi Province Center for Disease Control and Prevention gave permission to use data from the 2010 National Physical Fitness and Health Surveillance Survey. The survey had been conducted in 31 provinces (autonomous regions, municipalities) covering the whole of mainland China. However, the data used in this analysis pertains to one province: Guangxi province. The subjects were sampled by stratified cluster sampling method. In the first stage, five administrative areas were randomly selected from Guangxi. Then forty eight schools including primary, secondary, high school and university (college) were randomly selected from each selected area, ensuring proportionate representation of different economic levels and ethnic groups. Two classes from each grade from each selected school were randomly selected. All students in the selected classes were recruited as study subjects. A total of 19,859 students aged 7 – 22 participated in the national study. We aimed to assess the association of television viewing with caries in permanent teeth among adolescents. Therefore, only those aged 12, 14 and 17 years were included. A total of 3568 adolescents from Guangxi province were approached to participate. Ethical approval was obtained from the Ethical Committee and the Ministry of Public Health in China. Participants and their parents consented to participate in the study. Dental examinations were conducted by four experienced dental examiners. Examiners had been calibrated for diagnosing decayed teeth as part of the training of examiners for the national survey. Inter-examiner reliability was assessed and the kappa values were over 0.80 for all examiners. The WHO method for dental survey and criteria for diagnosing dental caries were used to conduct dental examination [19]. A questionnaire was administered to collect data on behavioural factors and demographic characteristics and were completed by the students themselves during the school time. The questionnaire included a question about time spent watching television per day: <30, 30–60, 60–120, 120–180 or >180 minutes per day.

### Statistical analysis

Stata software was used for the analysis. The number of untreated dental caries in permanent teeth, and DMFT score were the main outcomes. The main explanatory variable indicated the accumulated time spent watching television per day (<30; 30–60; 60/120; 120,180; >180 minutes/day). We also included variables pertaining to sex, age, ethnicity (Han, Zhuang and Yao). The survey did not include questions on oral hygiene or dietary habits about sugars consumption. Hence, questions on eating breakfast regularly and frequency of drinking milk were used as surrogate measures for dietary habits.

First, to describe the characteristics of the sample, the level of the disease and the distribution of explanatory variables within those with and without caries, we assessed the distribution of all explanatory variables within groups with and without caries experience. Two sets of negative binomial regression models were used to examine the association between TV viewing, and the mean number of decayed teeth and DMFT score. The rate ratio was assessed. It is the ratio between level of disease with no exposure and that after a unit increase in the exposure. The first model was adjusted for age, sex, ethnicity, and area of residence (urban/rural). The second model additionally adjusted for whether the child regularly ate breakfast and frequency of milk consumptions. The purpose of the modelling strategies was to assess whether accounting for the surrogate measures of dietary habits would alter the association between TV viewing and dental caries.

## Results

Of the 3568 students originally approached, 3,452 returned valid questionnaires. A response rate of 96.7%. Table 1 exhibits the distribution of all variables included in the analysis. The mean numbers of untreated caries in permanent teeth and DMFT were 1.56 (SD 2.34) and 1.84 (SD 2.66), respectively. The rate ratios of DMFT for those who watched television for 30–60, 60–120, 120–180 and >180 compared to those who spent <30 minutes/day were 1.03, 1.22, 1.25, and 1.28, respectively (Table 2). In other words the ratio of DMFT between those who viewed TV for less than 30 minutes and those who viewed TV for 30–60 minutes was 1.03. Similar relationship between the probabilities of having more untreated caries and time spent watching television was observed. Those who spent two hours or more watching television had a rate ratio of 1.26 compared to those who spent less than 30 minutes/day (Table 3). DMFT and decayed teeth were also significantly higher among females than males, adolescents living in rural than urban and Zhuang ethnicity than Han (Tables 2 and 3).

**Table 1:** **Distribution of explanatory variables within groups by caries experience**

		Percentage		
		Total	Within caries groups (DMFT = 1 or more)	
			Yes	No
Sex	Females	49.9%	55.1%	44.0%
	Males	50.1%	44.9%	56.0%
Age	12 years	32.8%	31.1%	34.6%
	14 years	33.2%	32.6%	33.9%
	17 years	34.0%	36.4%	31.5%
Ethnicity	Han	50.9%	45.1%	57.2%
	Zhuang	34.2%	46.1%	21.2%
	Yao	14.9%	8.8%	21.6%
Area of residence	Rural	57.7%	56.1%	59.5%
	Urban	42.3%	43.9%	40.5%
Watch TV	<30 minutes/day	36.0%	34.1%	38.0%
	30-60 minutes/day	25.0%	24.9%	25.2%
	60-120 minutes/day	21.3%	21.5%	21.1%
	120-180 minutes/day	9.3%	10.5%	8.0%
	>180 minutes/day	8.4%	9.0%	7.7%
Eat breakfast	Everyday	85.6%	86.6%	84.7%
	Less often	14.3%	13.4%	15.3%
Milk consumption	Never	12.6%	11.7%	13.6%
	Sometimes	65.1%	66.4%	63.6%
	Once/day	17.0%	16.8%	17.1%
	More than once/day	5.3%	5.0%	5.7%

**Table 2:** **Rate ratios of DMFT for 12–17 year olds and TV viewing, by age, sex, ethnicity and diet**

		Model 1	Model 2
		Rate ratio	Rate ratio
Age (reference: 12 years)	Age 14 years	1.15^*^ (1.02, 1.30)	1.14^*^ (1.01, 1.29)
	Age 17 years	1.43^***^ (1.27, 1.62)	1.43^***^ (1.27, 1.62)
Sex (female)		1.45^***^ (1.31, 1.60)	1.46^***^ (1.32, 1.61)
City (rural)		1.29^***^ (1.16, 1.43)	1.27^***^ (1.14, 1.42)
Ethnicity: (reference: Han)	Ethnicity Zhuang	1.84^***^ (1.65, 2.04)	1.84^***^ (1.65, 2.05)
	Ethnicity Yao	0.36^***^ (0.30, 0.43)	0.35^***^ (0.29, 0.42)
TV (reference: <30 minutes/day)	30-60 minutes	1.03 (0.90, 1.17)	1.04 (0.91, 1.18)
	60-120 minutes	1.22^**^ (1.07, 1.40)	1.23^**^ (1.07, 1.41)
	120-180 minutes	1.25^*^ (1.04, 1.49)	1.25^*^ (1.05, 1.50)
	>180 minutes	1.28^**^ (1.06, 1.55)	1.29^**^ (1.07, 1.55)
Drink milk (reference: never)	Sometimes		0.90 (0.77, 1.05)
	Every day		0.94 (0.78, 1.15)
	Twice a day		0.87 (0.67, 1.14)
Breakfast (Have breakfast most days)			0.96 (0.82, 1.12)

Model 1 adjusted for age, sex, area of residence (rural/urban), Ethnicity, TV viewing. Model 2 additionally adjusted for eating breakfast regularly, and frequency of drinking milk. ^***^ *p < 0.001*, ^**^ *p < 0.01*, ^*^ *p < 0.05.*

*Model 1: log likelihood chi-square: 415.73, Prob > chi-square:<0.001, Pseudo R-square: 0.0334.*

*Model 2: log likelihood chi-square: 418.24, Prob > chi-square:<0.001, Pseudo R-square: 0.0336.*

**Table 3:** **Rate ratio of decayed permanent teeth in 12–17 year olds and TV, by age, sex, ethnicity and diet**

		Model 1	Model 2
		Rate ratio	Rate ratio
Age (reference: 12 years)	Age 14 years	1.11 (0.98, 1.27)	1.09 (0.96, 1.24)
	Age 17 years	1.28^***^ (1.12, 1.45)	1.27^***^ (1.12, 1.45)
Sex (female)		1.40^***^ (1.26, 1.56)	1.42^***^ (1.28, 1.57)
City (rural)		1.49^***^ (1.33, 1.66)	1.47^***^ (1.31, 1.65)
Ethnicity: (reference: Han)	Ethnicity Zhuang	1.61^***^ (1.44, 1.80)	1.60^***^ (1.43, 1.78)
	Ethnicity Yao	0.32^***^ (0.27, 0.39)	0.31^***^ (0.26, 0.38)
TV (reference: <30 minutes/day)	30-60 minutes	1.04 (0.90, 1.19)	1.04 (0.91, 1.20)
	60-120 minutes	1.24^**^ (1.07, 1.43)	1.24^**^ (1.07, 1.43)
	120-180 minutes	1.26^*^ (1.04, 1.52)	1.26^*^ (1.04, 1.52)
	>180 minutes	1.26^*^ (1.04, 1.54)	1.26^*^ (1.04, 1.54)
Drink milk (reference: never)	Sometimes		0.91 (0.77, 1.07)
	Every day		0.94 (0.77, 1.17)
	Twice a day		0.84 (0.64, 1.12)
Breakfast (Have breakfast most days)			0.87 (0.75, 1.03)

Model 1 adjusted for age, sex, area of residence (rural/ urban), Ethnicity, TV viewing. Model 2 additionally adjusted for eating breakfast regularly, and frequency of drinking milk. ^***^ *p < 0.001*, ^**^ *p < 0.01*, ^*^ *p < 0.05.*

*For Model 1: log likelihood chi-square: 334.19, Prob > chi-square:<0.001, Pseudo R-square: 0.0289.*

*For Model 2: log likelihood chi-square: 338.70, Prob > chi-square:<0.001, Pseudo R-square: 0.0293.*

## Discussion

The findings of this study demonstrated a positive association between length of television viewing per day and dental caries among Chinese adolescents. While this association has been assessed in other countries [15, 20], it has not been examined before in a Chinese population. The major finding was that TV viewing time had a significant linear association with decayed teeth and the mean DMFT. The longer the time spent watching TV, the greater the number of decayed teeth and DMFT score. This relationship persisted even after accounting for some socio-demographic factors and dietary habits.

The underlying mechanisms of the association between TV viewing and dental caries are not yet fully understood. It has been shown that children and adolescents who watched television for longer were more likely to consume more sweetened beverages and snacks [10, 15]. And, as in the present study, Ghimire [15] reported a significant correlation between watching television advertisements and dental caries. Child TV viewers are exposed to messages that predominantly promotes unhealthy confections and drinks high in fats, sugars, and salt and other foods associated with obesity, dental caries, and other chronic diseases [21, 22]. About 50% of advertisements on children’s favourite channels were for cariogenic food and drinks. Among them, most advertisements were for chocolates and soft drinks [15]. Fruit, vegetables, protein-rich foods such as meat, fish, poultry, beans, nuts, eggs, and dairy products were rarely advertised, whereas foods rich in fats and sweets were advertised frequently, with candy being the most commonly advertised food [23]. Similarly, Rodd and Patel [13] found that 34% of the advertisements were related to food and drink products on children’s channels, 95% of these being deemed potentially cariogenic or erosive to teeth [13]. In China, viewing TV for longer was significantly associated with overweight among children and adolescents [12]. Giving the common risk factors between caries and obesity, it is plausible that a similar pathway links TV viewing and dental caries among Chinese children and adolescents [12]. In the current analysis, adjustment for some surrogate measures of dietary habits did not affect the association between TV viewing and dental caries, possibly because they do not reflect whether or not a child consumed cariogenic food.

A study investigating consumption of ten types of junk food in 1019 children and adolescent aged 8–16 years in Beijing found that the percentages eating biscuits, coke or similar drinks, cold and sweet food, barbecue food more than once a day were 32%, 27% and 37%, respectively [24]. Information on ‘junk’ food mainly came from sources such as advertisements on TV (67%), mothers (9%) and newspapers or magazines (6%) [24].

In contrast with studies in Western countries, in this study females were more likely to have dental caries than males, which was consistent with findings from an earlier national survey [5]. However, other studies in China did not find sex differences in dental caries [25]. One of the important findings of our analysis was the variation in dental caries between the ethnic groups, namely Han, Zhuang and Yao. Zhuang ethnicity children had significantly higher levels of dental caries than Hans. This finding was consistent with a recent review of ethnic variation in dental caries in China that reported that ethnic minorities, such as the Zhuang, had higher levels of dental caries than the Han ethnic group [25].

The findings of this study should be interpreted with caution given the cross-sectional design of the survey. The lack of data on oral health-related behaviours, and individual indicators of socioeconomic status is another limitation. However, the analysis was adjusted for ethnicity, area of residence (rural/urban) and for some dietary habits.

## Conclusions

This analysis demonstrated for the first time, an association between dental caries and TV viewing among adolescents in China. There was a higher probability of having more decayed teeth with increasing time spent watching television. The findings partially highlight the impact of the changing lifestyle of the Chinese population and its potential impact on oral health. Future research should examine the pathways linking television viewing and dental caries in the Chinese population.
